# Clinical use of thyroglobulin: not only thyroid cancer

**DOI:** 10.1007/s12020-023-03658-3

**Published:** 2024-01-06

**Authors:** Agata Berlińska, Renata Świątkowska-Stodulska

**Affiliations:** Department of Endocrinology and Internal Medicine, Faculty of Medicine, Medical University of Gdańsk, Gdańsk, Poland

**Keywords:** Thyroglobulin, Thyroid, Thyroiditis, Immune‐checkpoint inhibitors, COVID-19

## Abstract

Thyroglobulin (TG) is a dimeric glycoprotein produced exclusively by mature thyroid tissue and stored within the follicular lumen. It is essential for the organification of iodine and the production of thyroid hormones. The concentration of TG in the bloodstream varies between individuals and depends on factors such as thyroid mass, stimulation of the gland by thyrotropin or autoantibodies, and tissue destruction. TG is essential to monitor patients with differentiated thyroid cancer; however, its use is not limited only to this clinical entity. Measurement of circulating TG can provide better insight into numerous clinical scenarios, such as destructive thyroiditis, presence of ectopic thyroid tissue, thyroid trauma, factitious thyrotoxicosis, or iodine nutrition. Lately, TG has found its new clinical use in immune checkpoint-related thyroid dysfunction. TG measurement should be performed carefully in patients with antithyroglobulin antibodies due to possible laboratory interferences. In this review, we offer a summary of current knowledge about the clinical use of TG and the implications it brings to daily practice.

## Introduction

**Fig. 1:**
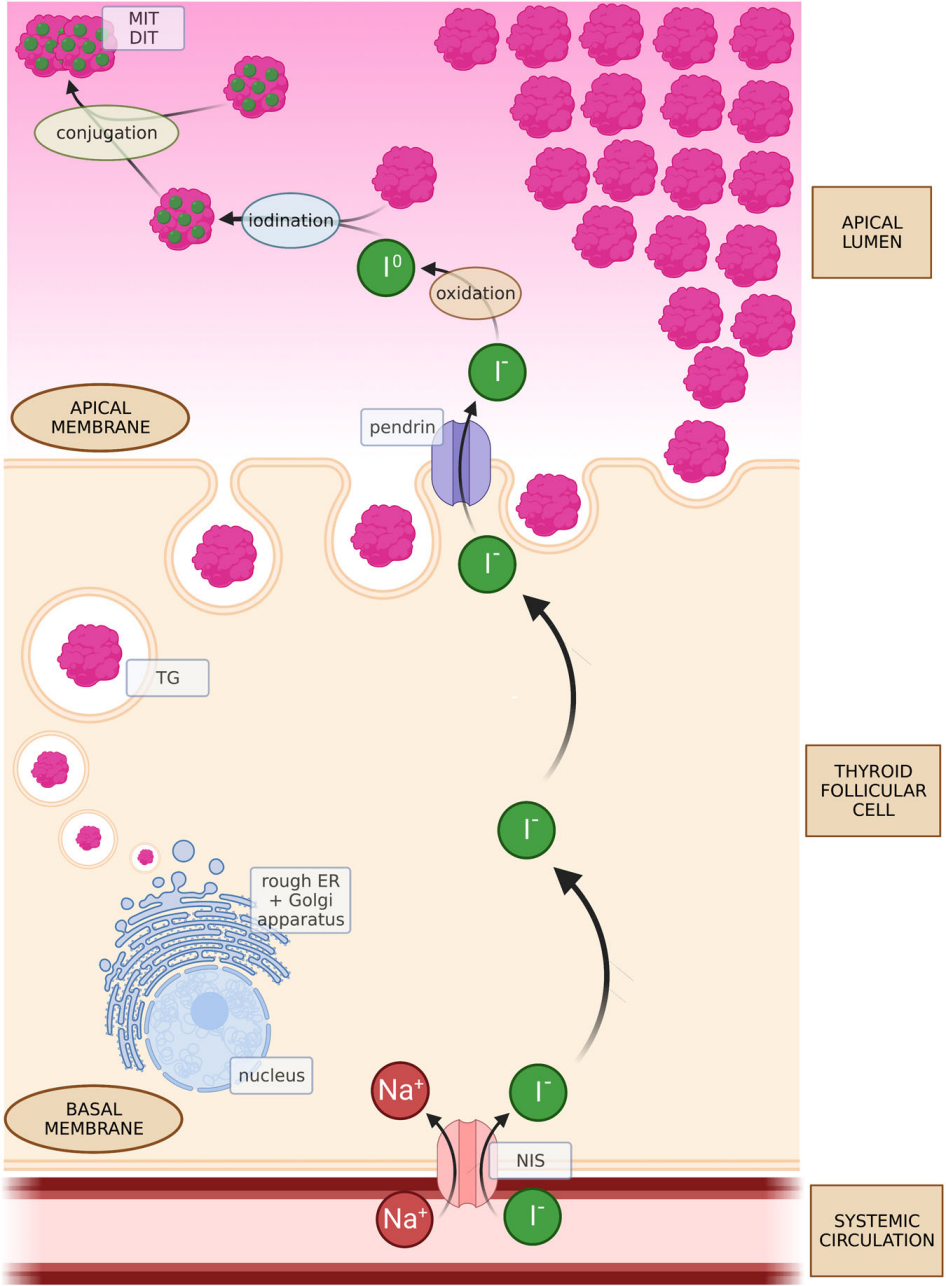
Synthesis of TG and its exocytosis into the apical lumen. Iodination and conjugation of TG. DIT diiodothyrosine, ER endoplasmic reticulum, MIT monoiodothyrosine, NIS sodium/iodide symporter, TG thyroglobulin. Created with BioRender.com

**Fig. 2:**
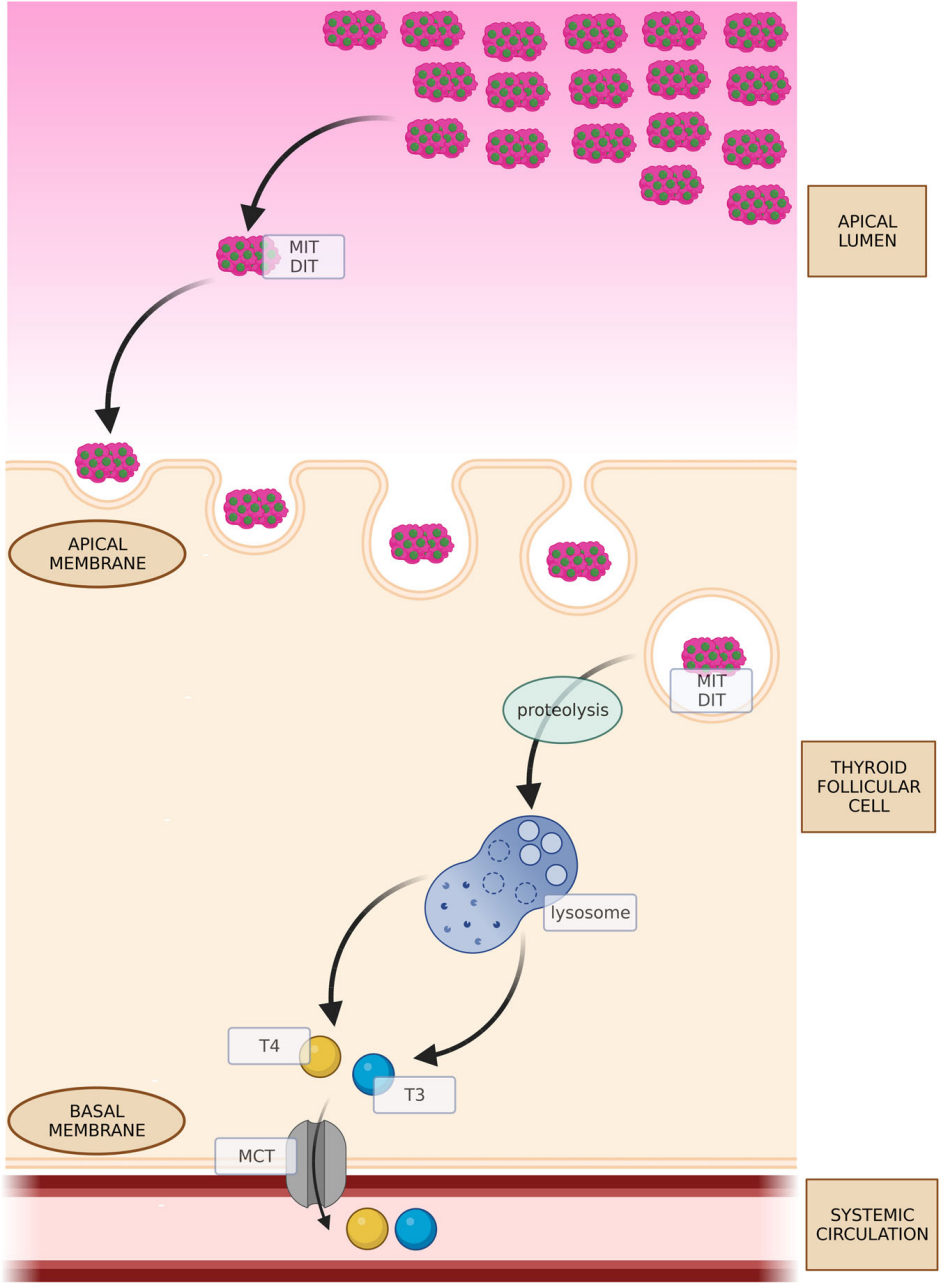
Endocytosis of TG from the apical lumen and cleavage of thyroid hormones. DIT diiodothyrosine, MCT monocarboxylate transporter, MIT monoiodothyrosine, T3 triiodothyronine, T4 thyroxine. Created with BioRender.com

Thyroglobulin (TG) is a 660 kDa dimeric glycoprotein produced exclusively by thyroid follicular cells and stored in the extracellular space of the thyroid, within the apical lumen. TG is crucial for the organification of iodine and is an organic matrix for the synthesis of iodothyrosines. The concentration of iodine in the bloodstream is low and its uptake in the form of iodide (I^-^) is an active process facilitated by the sodium/iodide symporter (NIS); later, pendrin promotes the transport of iodide to the colloid. Thyroid peroxidase (TPO) is responsible for hydrogen peroxide-dependent oxidation of iodide. After rapid oxidation, iodine is incorporated into TG (Fig. 1). Iodinated TG can form monoiodothyrosine (MIT) and diiodothyrosine (DIT), which are direct precursors of thyroid hormones. The coupling reaction between MIT and DIT results in the production of thyroid hormones: triiodothyronine (T3) and thyroxine (T4). After TSH-dependent stimulation, colloid TG undergoes endocytosis into thyroid follicular cells (Fig. 2). Within the cells, protein cleavage leads to the disengagement of thyroid hormones from TG. Thyroid hormones are then released into the bloodstream, where they can be bound to carrier proteins or remain free. TG is generally reabsorbed into the follicular lumen; however, under some conditions, it can be present in the bloodstream at elevated concentrations [1].

TG can be seen as a marker of quantity, activity, and destruction of thyroid tissue. Its concentration depends on stimulation by thyrotropin (TSH) or TSH receptor autoantibodies. Therefore, TG levels increase in the bloodstream in hyperthyroidism, goiter, thyroid cancer derived from follicular epithelial cells, thyroid trauma, or destructive inflammation [2–4]. On the other hand, TG might be below the detection threshold in factitious thyrotoxicosis or certain forms of congenital hypothyroidism when the TSH stimulation is lacking or thyroid tissue is absent. It was shown that circulating TG particles differ significantly according to their molecular size and properties between patients with subacute thyroiditis (SAT), Graves’ disease, and thyroid cancer, suggesting different mechanisms of TG release between these disorders, typically related to the mode of transport across the cell wall [5]. Although the concentration of circulating TG depends greatly on the various pathophysiological processes, it is possible for TG-specific laboratory assays to be interfered with by antithyroglobulin antibodies (anti-TG abs) resulting in false positive or false negative results. In our review, we present the less common aspects of TG in daily practice. Table 1 contains a summary of TG trends and use in different clinical scenarios.

**Table 1:** TG trends and use in different clinical scenarios

Clinical disorder	Subclass (if applies)		Typical TG trends
Thyroid cancer	Well-differentiated follicular and papillary cancer		↑ TG in malignant spread or recurrence; ↓ TG after successful treatment and uneventful follow-up (Detailed by ESMO and ATA guidelines)
SAT			↑ TG in destructive hyperthyroid phase; Possible ↓ TG in severe hypothyroidism
PPT			↑ TG in destructive hyperthyroid phase; Possible ↓ TG in severe hypothyroidism
Immune checkpoint-related thyroid disorders	Thyroiditis		↑ TG in destructive hyperthyroid phase; Possible ↓ TG in severe hypothyroidism
	Hyperthyroidism		↑ TG
	Secondary hypothyroidism due to hypophysitis		Possible ↓ TG; more clinical data necessary
Ectopic thyroid	Struma ovarii		↑ TG possible
	Other ectopic locations		
Goiter and hyperthyroidism	Nontoxic goiter		↑ TG possible
	Toxic nodular goiter		↑ TG
	Graves’ disease		↑ TG
Iodine nutrition	Schoolchildren		TG in dry blood spot can predict adequate iodine nutrition (WHO/ICCIDD/UNICEF recommended method)
	Adults		↓ TG with improvement of iodine nutrition; more clinical data necessary
Congenital hypothyroidism	Thyroid dysgenesis		↓ TG
	Thyroid dyshormonogenesis	*TG* gene mutations	↓ TG
		*TPO* gene mutations	↑ TG
		Pendred syndrome	↑ TG
Factitious thyrotoxicosis	Ingestion of levothyroxine/liothyronine formulations		↓ TG
	Ingestion of unpurified thyroid extracts		↑ TG
Laboratory interferences in TG measurement caused by anti-TG antibodies			↓ TG or ↑ TG, depending on the assay

*ATA* American Thyroid Association, *ESMO* European Society for Medical Oncology, *ICCIDD* International Council for Control of Iodine Deficiency Disorders, *PPT* postpartum thyroiditis, *SAT* subacute thyroiditis, *TG* thyroglobulin, *TPO* thyroid peroxidase, *UNICEF* United Nations International Children’s Emergency Fund, *WHO* World Health Organization

## Thyroid cancer

TG is a well-established marker used in the treatment of patients with differentiated thyroid cancer: follicular and papillary carcinoma, both of which originate from thyroid follicular cells. High-sensitivity TG tests allow early suspicion or recognition of recurrence or spread of the disease. Therefore, clinical guidelines highlight the usefulness of serum TG in adult and pediatric populations to stage, treat, and detect recurrence of differentiated thyroid cancer after radical treatment [6–8]. TG can be measured at baseline or after stimulation by recombinant human TSH. The suggested TG cut-off points differ depending on the chosen modality of treatment (thyroidectomy alone, thyroidectomy with radioactive iodine [RAI] ablation of the residual tissue, lobectomy). The European Society for Medical Oncology (ESMO) and the American Thyroid Association (ATA) concluded that a baseline TG concentration <0.2 ng/ml in patients after total thyroidectomy with or without RAI indicates an excellent response to treatment (no signs of persistent thyroid malignancy) [6, 7]. An excellent response in patients who underwent total thyroidectomy and subsequent RAI treatment can also be defined as TG < 1 ng/ml after stimulation with recombinant TSH [6, 7]. On the other hand, the response to treatment is considered biochemically incomplete if the baseline TG is ≥1 ng/ml or ≥10 ng/ml after stimulation with recombinant TSH in patients after total thyroidectomy and RAI, and if the TG is >5 ng/ml or continuously increases in patients treated with total thyroidectomy without RAI [6, 7]. Whenever TG is checked, it is crucial to concomitantly evaluate circulating anti-TG abs, as they can interfere with the assays and produce false results [6]. Although high-sensitivity TG is a staple of care in patients with differentiated thyroid cancer, its clinical use could be limited in certain scenarios, such as only partial removal of the gland or no RAI ablation. However, since the main objective of this article was not TG in thyroid cancer and numerous other reviews and clinical guidelines focus on the topic, we will refrain from further discussion.

## Destructive thyroiditis: subacute thyroiditis

SAT, also known as de Quervain’s disease or subacute granulomatous thyroiditis, is a destructive thyroiditis caused by immune activation following viral infection. Different viruses, such as mumps, rubella, adenoviruses, coxsackieviruses, echoviruses, or recently SARS-CoV-2, can precede the occurrence of SAT [9, 10]. The onset of SAT is usually delayed several days, if not weeks, after infection. In some cases, the disease can be recurrent and certain populations are at increased risk of developing SAT due to genetics. Human leukocyte antigens (HLA) B*35, HLA B*18:01, HLA DRB1*01, and HLA C*04:01 are known risk factors for the development of SAT [11]. The disease typically evolves through three different clinical phases: hyperthyroidism, hypothyroidism, and euthyroidism. The typical sequence of events includes gland destruction, uncontrolled release of thyroid content, negative feedback to the pituitary, thyroid insufficiency, and finally healing and spontaneous recovery of thyroid function in most patients. There is a considerable disproportion between circulating T3 and T4, with higher levels of T4, possibly because T4 is the main product of the follicular cells stored within the gland [12]. A study by Teixeira et al. in a small sample of SAT patients showed that serum TG was markedly higher in patients with SAT than in controls (149 ± 52 ng/ml vs. 10.5 ± 1.0 ng/ml) and that TG remained higher than in healthy subjects up to 4–5 months after the acute phase of the disease [13]. Madeddu et al. showed that TG was elevated in 92% of patients with newly diagnosed SAT and decreased after the introduction of glucocorticoids [14]. Similarly, Yamamoto et al. observed that patients receiving prednisolone showed lower TG concentrations than their salicylate-treated counterparts after 4 weeks of treatment; the authors suggested that the observed decrease in TG concentration could be the result of impaired intrathyroid hydrolysis of colloid due to glucocorticoid use [15].

As the collateral damage created by COVID-19 started to come to light, researchers began investigating possible relationships between thyroid function disorders and SARS CoV-2 infection [16–25]. COVID-19-related SAT, after being reported for the first time in 2020 by Brancatella et al., became a special interest [26]. Systematic reviews by Trimboli et al. and Christensen et al. provided concise information on COVID-19-related SAT and showed that the disorder predominantly affected young women [19, 21]. Trimboli et al. estimated that the median time between COVID-19 and COVID-19-related SAT was 30 days, and the synchronic occurrence of COVID-19 and SAT was present only in 12.5% of the analyzed cases [21]. TG in post-COVID-19 SAT can be markedly elevated [27]. Viola et al. reviewed 24 studies describing 69 cases of post-COVID-19 SAT—TG was checked in 19 patients and was high in all cases; at the same time, each patient reported typical clinical findings (neck pain) and 68 individuals who underwent thyroid function tests showed biochemical thyrotoxicosis [28]. Brancatella et al. compared clinical outcomes in post-COVID-19 and prepandemic SAT. Post-COVID-19 SAT cases were characterized by higher levels of fT4, CRP, and TG (155 vs. 60 μg/l) and more commonly associated with persistent hypothyroidism [29]. In-hospital studies on thyroid function in COVID-19 showed that TG elevation may be, in fact, not very common and rarely associated with thyrotoxicosis [23, 30]. A prospective study by Świątkowska-Stodulska and Berlińska et al. confirmed that an increase in TG during the initial 10 days of hospital stay due to COVID-19 was not common and a thyrotoxic pattern of thyroid hormones was even more rare [30]. TG levels were significantly lower in patients treated with glucocorticoids, tended to decrease over time, and did not correlate with inflammatory markers or thyroid function tests in a sufficient and repetitive manner [30]. In addition, Campi et al. suggested that the in-hospital finding of transiently decreased TSH accompanied by normal fT4, fT3 and TG might not reflect a hyperthyroid phase of thyroiditis, but rather result from generalized inflammation and its direct effect on enzyme activity and TSH secretion [22]. As SAT due to COVID-19 seems to be noticeably delayed, it is possible that in-hospital studies did not demonstrate it due to the insufficient observation time.

In summary, TG could play a supportive role in distinguishing SAT cases, including patients after COVID-19, especially if measured during the destructive phase and evaluated together with clinical findings and other biochemical abnormalities.

## Destructive thyroiditis: postpartum thyroiditis

Postpartum thyroiditis (PPT) is a transient disorder that usually occurs within a year after delivery, affects an estimated 7.2% of postpartum women, and is the result of autoimmune inflammation of the gland [31, 32]. Women with *HLA DR-3, HLA DR-4*, and *HLA DR-5* show an increased risk of developing PPT [32]. PPT is a destructive and self-limiting disorder that presents with hyperthyroid, hypothyroid, and/or euthyroid phases similar to those seen in SAT [31, 32]. The hyperthyroid phase in PPT usually occurs 3 months after delivery, typically with mild clinical signs and symptoms [32]. Hidaka et al. in their study aimed at distinguishing between recurrent Graves’ disease and PPT noticed that serial measurement of TG can be beneficial in determining the background of thyrotoxicosis: women with PPT experience a drastic increase in TG compared to their status before the onset of the disease [33]. It is not uncommon to see positive anti-TPO and/or anti-TG abs in women affected by PPT, and some patients could develop bispecific antibodies targeted against TPO and TG; however, anti-TPO abs are much more prevalent, and the exclusive presence of anti-TG abs is believed to be infrequent [34, 35]. In fact, some believe that PPT can be viewed as an exacerbated form of autoimmune thyroiditis [32]. Wang and Tang et al. noted the presence of anti-TPO and anti-TG abs as independent risk factors for PPT; at the same time, an extended follow-up in their study showed that women with positive anti-thyroid antibodies were less likely to be euthyroid 2 and 3 years postpartum [36]. A study in women with positive thyroid autoantibodies by Parkes et al. suggested that measuring TG three months after delivery could help recognize patients who develop PPT, as their TG levels were considerably higher compared to controls (31 μg/l in affected individuals vs. 3.3 μg/l in healthy controls and 5.8 μg/l in euthyroid controls with positive thyroid autoantibodies) [37]. In the same group of patients, TG levels during extended follow-up correlated well with the degree of lasting hypothyroidism defined by the lowest fT4 and the highest TSH, as well as the severity of ultrasound changes [37]. Therefore, TG measurement could be a practical additional tool in determining the diagnosis and prognosis in PPT.

## Immune checkpoint-related thyroid dysfunction

Immune checkpoint inhibitors (ICIs) are a group of potent drugs that serve as (neo)adjuvant treatment in modern oncology, utilizing native immunity to combat malignancy. There are three main groups of ICIs: programmed cell death 1 (PD-1) inhibitors, programmed cell death ligand 1 (PD-L1) inhibitors, and cytotoxic T-lymphocyte-associated protein 4 (CTL-A4) inhibitors. Numerous drugs from these groups were registered for clinical use: pembrolizumab, nivolumab, dostarlimab being examples of PD-1 inhibitors, avelumab, durvalumab, atezolizumab – PD-L1 inhibitors, and tremelimumab and ipilimumab—CTL-A4 inhibitors. The list of indications for the use of ICIs is still expanding; currently, disorders such as malignant melanoma, renal cell carcinoma, multiple types of lung cancer, urothelial carcinoma, Hodgkin’s lymphoma, and many others, were approved for treatment. At the same time, multiple adverse effects of ICIs were described, including thyroiditis, hypothyroidism, hyperthyroidism, hypophysitis, and adrenal failure.

While the group is highly effective and widely used in developed countries, it has a notable risk of immune-related adverse events (irAEs) [38]. IrAEs are likely to appear early during anticancer treatment, usually within the initial weeks or months; however, they can appear in a delayed pattern, even after 12 months of treatment [38, 39]. Endocrine irAEs most often involve thyroid dysfunction; analysis by Muir et al. showed the mean prevalence of thyroid-related irAEs to be up to 10.8% in patients treated in phase III clinical trials (ipilimumab – 4.7%, nivolumab – 8.8%, pembrolizumab 15.6%, ipilimumab + nivolumab – 16.0%, atezolizumab – 22.2%, durvalumab – 13.5%) [39, 40]. Interestingly, the presence of thyroid irAEs could be tied with better prognosis in patients receiving ICIs [41, 42]. The exact background of thyroid-related irAEs is not completely clear and understood, likely developing as a result of complex crosstalk between genetic predispositions, humoral immunity, and cellular immunity [43]. However, it is hypothesized that it might be a form of painless thyroiditis that progresses through various clinical stages and produces a typical spectrum of detected abnormalities [38, 40]. The assault on thyroid tissue results in a destructive self-limiting hyperthyroid phase, which later progresses to hypothyroidism and eventually euthyroidism – a pattern similar to SAT and PPT. It is noteworthy that some patients might never recover proper thyroid function and remain permanently hypothyroid. Thyroid function abnormalities and their clinical presentation are often mild and only observed incidentally during follow-up; however, some patients can develop typical signs and symptoms of hypo- or hyperthyroidism, sometimes overshadowed by manifestations of malignancy [39, 40]. Kurimoto et al. found that an early increase in serum TG and anti-TG abs (≤4 weeks of ICI treatment) is helpful in distinguishing the patients who will and who will not develop thyroid disorders related to ICIs; both TG and anti-TG abs are higher in patients experiencing specific irAEs [44]. According to the same research, other factors, such as baseline serum granulocyte-macrophage colony-stimulating factor, interleukin 1β, and interleukin 2, are more elevated in patients with irAEs compared to controls without irAEs [44]. Some irAEs can become chronic, with rheumatologic and endocrine manifestations the most common [38]. Alhusseini et al. presented a series of patients developing immunotherapy-related hypothyroidism: TG was elevated in exactly half of the patients during the hyperthyroid phase, 40% had anti-TG abs, and 80% were hypothyroid at 6 months of observation [45]. A study by Inaba et al. showed that TG levels ≥33.7 ng/ml were significantly more prevalent in patients who developed permanent thyroid dysfunction and required continuous treatment than in those who needed only transient therapy [46]. Although primary hypothyroidism is a common irAE, a more rare form, central hypothyroidism, can develop due to pituitary dysfunction in the form of hypophysitis. In fact, as reported by Jessel et al., secondary hypothyroidism was among the most common manifestations of hypophysitis (35% of cases), together with adrenal insufficiency and hypogonadism [47]. As central hypothyroidism is the result of the lack of TSH signaling from the anterior pituitary and not destruction of the thyroid gland, it should not lead to excessive TG release into the bloodstream. On the contrary, if TSH stimulation decreases, TG levels could be expected to drop, as TSH is one of the factors that facilitate thyroid hormone coupling and TG release. Given all the above and knowing the destructive history of immunotherapy-related thyroiditis, TG can be a useful clinical parameter that allows appropriate treatment and long-term care in patients treated with ICIs. It could allow for the early detection, diagnosis, and continuous clinical monitoring of possible complications, as well as be useful in distinguishing between the secondary and primary background of hypothyroidism.

## Thyroid trauma

Physical trauma to the thyroid gland could trigger the release of thyroid hormones and TG from damaged tissues, which was already reported in the 1960s [48]. A recent study by Senese et al. confirmed that intraoperative manipulation of the thyroid gland leads to noticeable changes in the functional parameters of the thyroid [49]. According to Senese et al., patients who did not receive any treatment that interfered with thyroid function showed a significant increase in T4 and TG during surgery, a trend that later reversed, with a noticeable reduction in TSH, T4, T3, and TG levels after surgery [49]. Interestingly, patients with Graves’ disease tended to exhibit significantly higher TG concentrations both intraoperatively and postoperatively, highlighting the complex and multifactorial nature of TG release [49]. A marked increase in TG concentration, sometimes as high as 7000 ng/ml, could be seen 24–48 h after thyroidectomy or radioiodine ablation and did not necessarily come with high peripheral thyroid hormones [50]. A study by Rudofsky et al. in 40 patients parathyroidectomized due to secondary hyperparathyroidism showed biochemical indicators of hyperthyroidism in 77% of the analyzed patients; at the same time, there was a significant increase in TG levels, a parameter proposed by the authors as a predictive indicator of transient thyrotoxicosis in this group [51]. TG concentration had a significant positive correlation with fT4 and fT3 and negative with TSH [51]. The abnormalities were likely caused by physical manipulation of the gland during surgery and could also be referred to as “palpation thyroiditis”. In a study by Rudofsky et al., all thyroid parameters returned within the normal range after 40 days [51]. Madill et al. shared a case report of a young woman with transient thyrotoxicosis after parathyroidectomy with similar findings – the disorder resolved spontaneously in 14 days [52]. Serum TG can peak after much smaller invasive procedures, such as fine needle biopsy of the thyroid or lymph nodes suspected of neoplastic spread [53–55]. Therefore, in the setting of procedures involving manual or instrumentary thyroid manipulation, it is likely that TG concentration temporarily increases, often together with peripheral thyroid hormones, and returns to the baseline spontaneously. Physicians should be aware of these transient alterations to be able to provide appropriate care and support to patients.

## Struma ovarii and ectopic thyroid

Struma ovarii (SO), also known as ovarian goiter, is a mature teratoma of the ovary that contains predominantly (>50%) or exclusively thyroid tissue [56]. Thyroid tissue can be detected in 5–20% of mature teratomas [56]. SO can be a rare cause of thyrotoxicosis, with up to 8% of tumors overproducing thyroid hormones [57–60]. Like thyroid tissue in a normal anatomical location, SO can undergo a malignant transformation. A systematic review of 144 cases by Cui et al. showed that papillary thyroid carcinoma was the most prevalent neoplasm that occurred in SO (50% of cases) and more than 50% of the analyzed patients had extraovarian metastases, most commonly within the pelvis [61]. More than half of the affected individuals showed elevated Ca125 which could make the diagnosis difficult, as Ca125 is traditionally perceived as a marker of ovarian cancer [61]. The immunohistochemical examination of SO should be positive for TG, which is typical of the presence of mature follicular thyroid tissue [61–63]. In some cases of SO, circulating TG is markedly elevated, sometimes raising concerns about a possible spread of well-differentiated thyroid cancer [64–66]. In addition, if SO is itself malignant and metastatic, TG increases as is usual for cancer derived from thyroid follicular epithelial cells [61]. The main modality of treatment for SO involves surgery. Radiotherapy, chemotherapy, and/or RAI can be introduced in some cases, usually if malignant spread is suspected [61, 67, 68]. On rare occasions, thyroid tissue can be found in other ectopic locations often related to the developmental tract of the thyroid gland: base of the tongue, lateral neck, submental area, mediastinum, axilla, trachea, gastrointestinal tract, adrenals, or even iris [69, 70]. As in SO, the development of cancer is possible; however, it seems rare for ectopic thyroid tissue in the head and neck area, and papillary thyroid cancer seems to be the most prevalent in such cases [71, 72]. Ectopic thyroid, although rare, may be a surprising and confusing finding, in some cases reiterating concerns about the recurrence or spread of well-differentiated thyroid cancer. SO’s products can resemble those of a regular thyroid gland, including the ability to release TG. In ambiguous cases, radiological studies such as pelvic magnetic resonance/computed tomography and iodine scintigraphy could allow verification of a suspected SO [73].

## Goiter and thyrotoxicosis

An abnormally enlarged thyroid gland is termed “a goiter”. Goiters are a heterogeneous group and can be associated with different endocrine abnormalities, such as hyperthyroidism (Graves’ disease, toxic nodular goiter), hypothyroidism (Hashimoto’s thyroiditis, endemic goiter), or remain euthyroid (nontoxic nodular goiter). On rare occasions, goiters can result from anterior pituitary adenomas that produce TSH or resistance to thyroid hormones [74, 75]. It seems that different types of goiters should follow similar pathophysiological paths leading to increased circulating TG: general increase in thyroid mass and, sometimes, hyperactivity of the gland.

Torrigiani et al. noticed that patients with larger goiter had higher circulating TG than those with smaller goiter and that TG was more elevated in patients who were thyrotoxic than in those who were not [48]. Levine et al. showed that basal TG levels increased above 100 ng/ml in a third of patients with non-toxic goiter, but no correlation with the goiter size could be found; similar data were reported by Pezzino et al. [76, 77]. Despite the clear methodological limitations of these classic endocrine studies from the 1960s and 1970s, they can be perceived as successful attempts to correlate thyroid function and size with circulating TG. TG can be increased in patients with non-toxic endemic or sporadic goiter, which can reflect not only hyperstimulation by TSH and glandular mass but, as some authors postulate, also other factors, such as hypoiodination of TG and intrathyroid necrosis leading to uninhibited release of tissue content [77–79]. Rink et al. confirmed that TG levels in the nodular goiter—an entity known to contain occasional areas of spontaneous necrosis—can be higher than could be assumed based on the size of the goiter alone [80]. Ericsson et al. observed that serum TG was elevated at baseline in most patients with thyrotoxicosis of various origin; however, no clear correlation was found with post-treatment TG levels or treatment results [81]. Based on the data from the same study, there was a higher chance of relapse in patients who showed a higher serum TG prior to treatment [81].

In Graves’ disease, the thyroid is not only considerably enlarged, but is also constantly hyperstimulated by antibodies that target the TSH receptor. Rink et al. observed that TG was significantly more elevated in Graves’ disease than in diffuse goiter, but almost half of the acquired results still fit within the laboratory norm [80]. Due to the wide spectrum of autoantibodies activity, Graves’ disease can manifest itself not only as a diffuse thyrotoxic goiter, but can affect other organs, such as the skin, connective tissue, or ocular apparatus. Khamisi et al. postulated that TG could be a warning sign of Graves ophthalmopathy (GO), as baseline TG was significantly higher in patients who developed GO compared to those who did not [82]. The authors suggested that circulating TG could represent the magnitude of stressor that affects not only the thyroid, but also the retroorbital tissues [82].

A study in patients with toxic nodular goiter, multinodular toxic goiter, and toxic thyroid adenomas referred for radioiodine therapy presented by Bonefačić et al. showed that TG can be viewed as a marker of response to radioiodine ablation [83]. A significant decrease in circulating TG was observed in patients who were able to reach euthyroidism or hypothyroidism in the first 12 months after treatment; in patients with persistent hyperthyroidism, the basal TG was higher and did not show a tendency to decrease adequately after ablation [83].

## Iodine nutrition

Iodine nutrition is a difficult and complex topic and uniform data allowing straightforward recommendations is often lacking. Iodine status can be reflected not only in the concentration of TSH, T4, and urinary iodine excretion, but also in serum TG [84–86]. In 1994, a joint statement by the World Health Organization (WHO), the International Council for Control of Iodine Deficiency Disorders (ICCIDD), and the United Nations International Children’s Emergency Fund (UNICEF) suggested that circulating TG < 10 μg/l can be taken as a biomarker of adequate iodine intake in schoolchildren; however, serum TG did not appear in further recommendations [87]. Zimmermann et al. studied TG in dry blood spots of 700 schoolchildren, finding the designated norm to be between 4 and 40 μg/l [88]. In 2007, WHO/ICCIDD/UNICEF proposed a cut-off value for the dry blood spot test in schoolchildren with sufficient iodine intake to be 4–40 μg/l, while it did not indicate any values for serum TG [89].

Relatively higher TG can appear in populations deficient in iodine; however, both excess and deficiency of iodine were shown to present with an increase in TG [79]. Thyroid volume correlates positively with circulating TG in iodine-deficient populations [90]. The results of twin cross-sectional studies published by Vejbjerg et al. proved that iodization programs in iodine-deficient populations resulted in a significant decrease in mean TG regardless of sex and age of the recruited subjects, as well as led to a significant decrease in the number of cases that exceeded the predefined upper normal limit of circulating TG (11.3 vs. 3.7) [91]. Furthermore, the study showed a higher efficacy of serum TG compared to thyroid volume evaluated on ultrasound to predict iodine status [91]. A double-blind randomized placebo-controlled trial in adults with mild iodine deficiency by Ma et al. showed that an improvement in urinary iodine concentration was associated with a drop in circulating TG [92]. A cross-sectional study by Du et al. confirmed that TG can serve well as a marker of iodine nutrition in adults, but also warned about the possible impact of thyroid disorders on circulating TG and the possibility of false results leading to erroneous clinical conclusions [79]. Dineva et al. assessed TG as a marker of iodine status in pregnant women, finding a link between urinary iodine concentration and serum TG, which appeared especially strong in iodine deficient women. Based on this study, TG could be viewed as a complementary indicator of iodine sufficiency [93]. A single-blind randomized placebo-controlled trial by Censi et al. brought similar results [94]. However, to this day, the universal WHO recommendations do not opt for routine TG measurement in adults, including pregnant women, suggesting urinary free iodine instead [89].

Importantly, isolated serum TG measurement may not be an optimal assessment option in individuals with anti-TG abs due to the possibility of interference with the assay and in individuals with thyroid disorders that lead to unphysiological TG release [79, 90]. However, TG could eventually become a simple and inexpensive method of evaluating iodine status in the general population. The available data hint at its high clinical usefulness in all age groups, but solid clinical data is necessary in groups other than schoolchildren. Interestingly, simple methods, such as measuring TG in dry blood spots, can be seen as promising tools for assessing iodine nutrition, as shown in an example of children [87, 95–98].

## Congenital hypothyroidism

Congenital hypothyroidism is a heterogenous group of disorders that result in a hypofunctioning thyroid in a newborn. Since screening programs in newborns were introduced, many cases of hypothyroidism are diagnosed early and levothyroxine supplementation can be introduced to prevent developmental complications. Typically, the screening procedure is based on the measurement of TSH or T4 in the dry blood spot [99, 100]. The TG assessment can be helpful in determining the background of congenital hypothyroidism, as TG concentration differs between various scenarios.

Thyroid dysgenesis is rare and can manifest as thyroid ectopy (the most common form – 2/3 cases), thyroid hypoplasia, and athyreosis [101]. Thyroid dysgenesis appears to occur sporadically; however, certain familial patterns can be observed [101]. At the same time, thyroid dysgenesis can be associated with mutations in the *PAX-8, TTF1, TTF2*, and *NKX2.5* genes [101, 102]. A study by Muir et al. showed that infants classified as athyrotic had a considerably lower TG concentration compared to children born with a goiter or ectopic thyroid; in the same study, the TG concentration was the highest in children with goiter (mean TG values: goiter – 149.1 pmol/l; ectopic thyroid – 60.5 pmol/l; athyrotic – 7.9 pmol/l) [101, 103]. However, it was suggested that if true thyroid agenesis was present, circulating TG could be unmeasurably low a few weeks after delivery [101]. In addition to serum thyroid hormones and TG, thyroid ultrasound and/or radionuclide uptake can facilitate the diagnosis [101, 104]. If radionuclide uptake is negative and TG can be measured in the circulation, a mutation that inactivates TSH receptors can be suspected [101].

Thyroid dyshormonogenesis is a complex topic as multiple steps can be affected. The main examples include pendrin, NIS, TG, and thyroid peroxidase [105]. Dyshormonogenesis due to altered TG synthesis results from mutations in the *TG* gene, and the circulating TG level is generally low [105, 106]. In thyroid peroxidase mutations (*TPO* gene), TG concentration tends to be elevated [105]. Mutation in the *SLC26A4* gene responsible for pendrin production leads to the development of an interesting clinical entity known as Pendred syndrome, which is characterized by bilateral sensorineural hearing loss and the development of euthyroid or hypothyroid goiter. A study of 17 unrelated patients with Pendred syndrome conducted by Friis et al. detected an increase in TG concentration in 13 of the individuals studied and a similar trend could be seen in most cases evaluated by other authors [107, 108].

As congenital hypothyroidism arises from various backgrounds, a thorough evaluation remains the key to establishing a proper diagnosis. As TG concentration typically differs between the main entities that cause congenital hypothyroidism, it can be a helpful diagnostic step that allows a complete investigation.

## Factitious thyrotoxicosis

Factitious disorder, also known as Munchausen syndrome, is a psychiatric disorder that causes people to intentionally fake an illness. It is usually imposed on self; however, it can be imposed on another (factitious disorder by proxy), often targeting children [109]. It is difficult to unanimously explain the motivations behind the factitious disorder; nevertheless, many professionals agree that it could be traced back to a profound need for medical attention or even tricking medical professionals [110]. A systematic review of 455 cases of factitious disorder carried out by Yates and Feldman showed that endocrine disorders were fabricated the most frequently (59 cases, 12.97%), with factitious hypoglycemia and hypercortisolemia reported the most common, but factitious thyrotoxicosis coming third with eight described cases [110]. A similar systematic review by Caselli et al. included 514 cases, among which 29 (5.6%) were endocrine-related and factitious thyrotoxicosis was observed in two patients [111]. Both systematic reviews showed that factitious disorder was more common in women who often had a history of mental illness [110, 111]. However, the case series of 49 patients with a factitious disorder presented by Bérar et al. did not include any cases of thyrotoxicosis, which only highlighted the irregularity and unexpectedness of the disorder [112]. In general, thyrotoxicosis is not difficult to fabricate, as thyroid hormone preparations are readily available and easily accessible. Exogenous abuse of thyroid hormones will lead to the development of signs and symptoms typical of thyrotoxicosis, as well as laboratory alterations typical of primary thyrotoxicosis: low TSH, high fT4 and/or fT3. Ingestion of excessive doses of exogenous thyroid hormones provokes potent negative feedback for the pituitary and endogenous TSH-derived thyrocyte activation of thyrocytes diminishes. As a result, TSH-stimulated endogenous release of thyroid hormones and concomitant leakage of TG can significantly decline. This was supported by observed cases of factitious thyrotoxicosis in which patients had low TSH, elevated fT3 and fT4, but low or nearly low TG [113–117]. Some patients choose less conservative substitution options, such as dessicated thyroid extracts that contain complete thyroid tissue and that are often difficult to standardize dose-wise and unpurified. In cases of overdose of these specimens, it is possible for the TG of preparations to peak in the bloodstream and be detected by conventional laboratory assays, which can cause considerable distress, especially in patients thyroidectomized due to thyroid cancer [118]. Therefore, however difficult that might be in factitious disorder, proper history-taking remains the key to solving the cases. Treatment of factitious thyrotoxicosis can be challenging, as it depends primarily on psychological interventions that require the full consent and cooperation of the patient. Unfortunately, patients with a factitious disorder often migrate to other medical facilities in the hope of finding unnecessary and often harmful treatment elsewhere.

## Laboratory assays

TG is usually assessed using radioimmunossay (RIA) or various immunometric methods (IM), such as, for example, the immunochemiluminescence assay or the immunoradiometric assay [87]. The measured TG concentration can vary depending on the chosen method, and the differences between tests can reach 65% in healthy individuals [87]. Anti-TG abs can interfere with assays, giving false concentrations of TG. In the presence of anti-TG abs, IM can measure TG falsely low, which can have detrimental consequences for patients with a history of thyroid cancer [119–121]. Therefore, it is suggested that anti-TG abs should be measured simultaneously with TG [121]. At the same time, the RIA method appears to be more resistant to alterations caused by anti-TG abs, but it can still produce falsely elevated and decreased TG results, depending on the reagents used for the assay and the patient-specific anti-TG abs [121, 122]. Therefore, patients who produce anti-TG abs require careful consideration and meticulous examination whenever it is necessary to assess TG levels. Novel diagnostic biomarkers that are independent of laboratory interference and allow the detection of differentiated metastatic thyroid cancer are in development, and serum midkine is one of the examples [123].

## Summary

TG is a valuable but, as it seems, notoriously underestimated clinical marker. The role of TG is not limited to the follow-up of differentiated thyroid cancer. Due to its physiological function and anatomical location, the circulating TG concentration can change in different clinical scenarios, from undetectable low in congenital athyreosis to extremely high in destructive thyroiditis. The concentration of TG concentration can vary due to laboratory interferences. Understanding the role of TG and its varying levels in the bloodstream can help diagnose and make decisions in multiple disorders; therefore, physicians should be aware of such clinical implications.
